# Case Report Associated with Aspergillosis and Hepatitis E Virus Coinfection in Himalayan Griffons

**DOI:** 10.1155/2015/287315

**Published:** 2015-10-28

**Authors:** Heng Li, Rining Zhu, Ruiping She, Chenglin Zhang, Ruihan Shi, Wei Li, Fang Du, Qiaoxing Wu, Fengjiao Hu, Yang Zhang, Majid Hussain Soomro, Changming Zheng

**Affiliations:** ^1^Laboratory of Veterinary Pathology and Public Health, College of Veterinary Medicine, China Agricultural University, Beijing 100193, China; ^2^Beijing Zoo, Beijing, China

## Abstract

This study involved a death which occurred in four Himalayan griffons housed in Beijing zoo, China. Based on pathogen identification and the pathological changes observed, we did characterize the fungi and Hepatitis E virus (HEV) in four dead Himalayan griffons. Pathological changes were severe. Membranous-like material was observed on the surface of the internal organs. Spleen was necrotic. Focal lymphocyte infiltration in the liver and many sunflower-like fungi nodules were evident in the tissues, especially in the kidney. PCR was used to identify the pathogen. Based on the 18SrRNA genomic sequence of known fungi, the results confirmed that all four dead Himalayan griffons were infected with *Aspergillus*. At the same time the detection of HEV also showed positive results. To the best of our knowledge, this work appears to be the first report of concurrent presence of Aspergillosis and Hepatitis E virus in rare avian species.

## 1. Introduction

The Himalayan griffon* Gyps himalayensis* is one of the resident species of the Himalayan and is the largest predator which eats carrion on the plateau. In the past twenty years, the numbers of Himalayan griffons declined sharply [1]. Rare literature regarding Himalayan griffon disease was reported before [2], especially on Aspergillosis and HEV coinfection.

Aspergillosis is a fungal infection caused by the genus* Aspergillus* affecting mostly the respiratory tract of humans and animals, with a higher occurrence in birds than in mammalians [3]. It infracts the respiratory system of many kinds of birds, leading to a range of disease manifestations from acute to chronic infections [4]. Aspergillosis has been reported in a wide variety of domesticated avian species like chickens [5], mallards [6], turkeys [7], geese [8], quails [9], and ostriches [10]. Nowadays, it has become a major hazard and has turned into a serious bird problem.

Hepatitis E (HE) is another epidemic disease. According to the literature reported, HEV is mainly prevalent in developing countries in Asia, Africa, and Latin America [11]. There are at least four genotypes of HEV, with genotypes 3 and 4 known to infect animals, and thought to be zoonotically transmitted [12].

Infection of birds with multiple pathogen is common; however, coinfection involving Aspergillosis and HEV has rarely been reported, especially in rare birds. Both of these two pathogens could lead to the zoonotic disease [13]. Hence, the objective of our study was to analyze the cause of death of the four Himalayan griffons and provide a new perspective for clinical research.

## 2. Materials and Methods

### 2.1. Ethics Statement

The animal experiments were approved by the Animal Care and Use Committee of China Agricultural University (CAU) (permit number: 20140310-059). We followed the guidelines of the CAU Animal Care and Use Committee in handling the experimental animals during this study.

### 2.2. Medical History

From March 6 to 7, 2014, an unknown disease occurred at the Beijing zoo, China. All of the four Himalayan griffons in the zoo were dead. No other obvious symptoms were presented by X-ray examination and blood examination before these animals were purchased from a farm in Xinjiang (87.68°E, 43.77°N) in November 2013. These four eight-year-old birds were all female and weighed 6 kg [normal adult griffons weigh 8–12 kg]. Before their death, they were found weak, emaciated, showing signs of dyspnea and incoordination, and unable to fly. The results of bacteria, Avian Influenza (FLU), and Newcastle Disease Virus (NDV) check were all negative.

### 2.3. Sampling and Pathological Examination

Necropsies were performed on four dead Himalayan griffons. The tissues examined included the heart, liver, spleen, lung, kidney, intestine, and air sac. All tissues used for histopathological examination were fixed in 2.5% (w/v) glutaraldehyde-polyoxymethylene solution for 48 h. The fixed tissues were routinely processed, embedded in paraffin, sectioned (4 *μ*m thickness), and stained with hematoxylin and eosin stain, Periodic Acid-Schiff stain, and Immunohistochemistry. Portions of the liver, lung, and kidney were used for pathogen detection and stored at −80°C for determination of pathogen.

### 2.4. Determination of Pathogen

According to the gross and histopathological lesions, five suspected pathogens were detected. Total RNA and DNA were extracted from liver, lung, kidney, heart, and spleen specimens using the UltraPure RNA Kit and the General AllGen Kit (CWBIO, Beijing, China), according to the manufacturer's instructions. The extracted RNA was used in reverse-transcription polymerase chain reaction (PCR) assays to detect Hepatitis E virus (HEV), Avian Influenza (FLU), and Newcastle Disease Virus (NDV). The extracted DNA was used to detect fungus. The primers used in this study are listed in Table 1. PCR for fungus included initial denaturation at 95°C for 3 min, followed by 35 cycles of 94°C for 1 min, 56°C for 1 min, and 72°C for 2 min, with a final extension step at 72°C for 10 min. Nested PCR was carried out to amplify a partial fragment of ORF2 (nt 5,983–6,349) of the HEV genome. The amplification parameters for both rounds of PCR were the same: 95°C for 7 min, followed by 35 cycles of 94°C for 1 min, 42°C for 1 min, and 72°C for 2 min, with a final extension step at 72°C for 10 min. Sterile ddH_2_O (1 *μ*L) was included as the negative control.

## 3. Results

### 3.1. Gross Lesions

**Figure 1:**
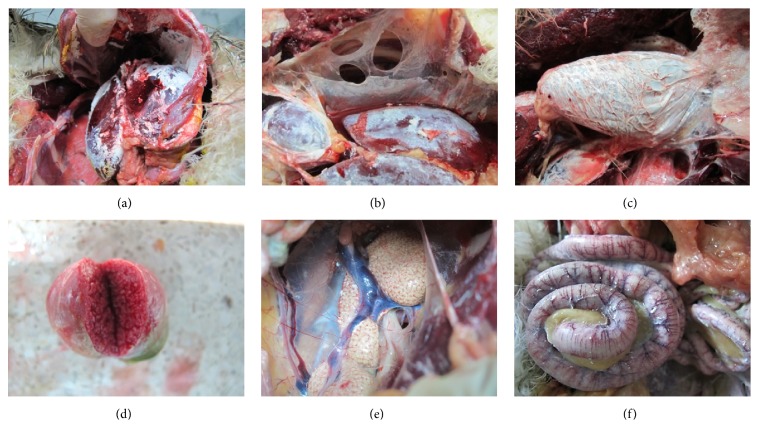
Necropsy observations. (a) Internal organs. (b) White liver and air sac. (c) Thick pericardium. (d) Spotted spleen. (e) Enlarged kidney. (f) Congestive vessel of the intestine.

Four dead Himalayan griffons were necropsied and diagnosed. Much white membranous-like material was observed on the surface of the internal organs (Figure 1(a)). Many whitish patches and spots were present in large areas of the liver and air sac (Figure 1(b)). The pericardium was white and thick, looking like the armored heart (Figure 1(c)). Small whitish nodules were observed on the sections of spleen (Figure 1(d)). Kidney became enlarged (Figure 1(e)) and intestinal vessels were engorged obviously (Figure 1(f)).

### 3.2. Histological Lesions

**Figure 2:**
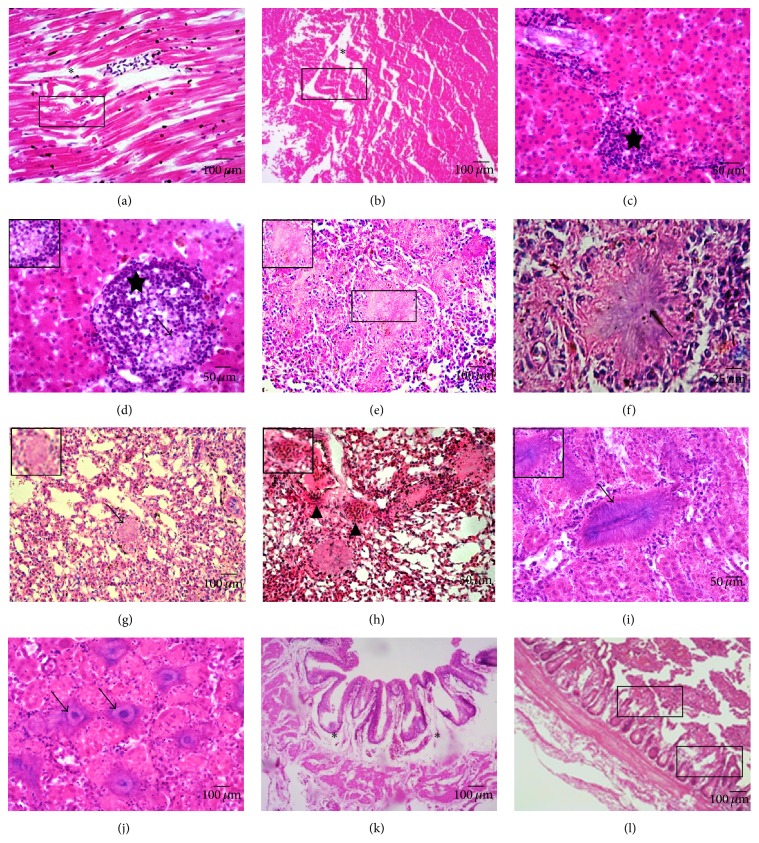
Histological lesions in multiple organs. Pathological changes were characterized by degeneration, edema, inflammatory infiltration, necrosis, and appearance of the flower-like fungi nodes. Heart (a, b): necrosis, edema, and wave-like degeneration in the cardiac muscle fiber. Liver (c, d): liver exhibiting hepatic necrosis and lymphocyte infiltration. Spleen (e, f): necrosis and fungi nodes in the spleen. Lung (g, h): lung with hemorrhage and fungi nodes. Kidney (i, j): many sunflower-like fungi nodes in the kidney. Intestine (k, l): edema and abruption of intestinal villi.

Pathological changes in various tissues were determined with microscopy. Examination of the heart demonstrated necrosis (karyolysis and pyknosis) (rectangle), fragmentation, and edema (emergence of cardiac muscle fiber gap) (asterisk) of the myocardium (Figure 2(a)). The cardiac muscle fiber was characterized as wave-like degeneration (morphological changes of cardiac muscle fibers) (rectangle) (Figure 2(b)). Focal lymphocyte infiltration (emergence of inflammatory cells) (star), particularly in the portal area, was observed in the liver (Figures 2(c) and 2(d)). A big number of hyphae had infiltrated in the central veins and were presented as clumps clearly (arrow) (Figure 2(d)). In the spleen large areas of tissues were necrotic (karyolysis) (rectangle) (Figure 2(e)), and many actinomorphous fungi nodes were seen distinctly (arrow) (Figure 2(f)). Examination of the lungs demonstrated large areas of hemorrhage (erythrocyte infiltration) (triangle), and fungi nodes infiltration (arrow), with very little normal histological structure (Figures 2(g) and 2(h)). A nephritic examination revealed that there were many sunflower-like fungi nodes existing in renal interstitium (arrow) (Figures 2(i) and 2(j)). Rupture of the intestinal villus was observed. The lamina propria of the intestinal villus showed necrosis (fracture of intestinal villi) (rectangle), and edema (broadening of interbowel) was present in the submucosal layer (asterisk) (Figures 2(k) and 2(l)). The main pathological changes observed in the various organs of the four Himalayan griffons are summarized in Table 2.

### 3.3. Periodic Acid-Schiff Stain and Immunohistochemistry

**Figure 3:**
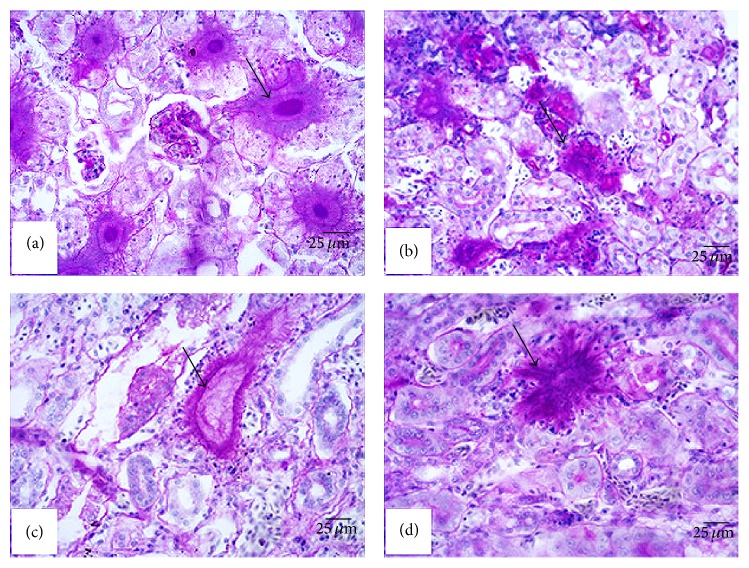
Detection of fungi in the kidneys of the four dead Himalayan griffons. (a) Positive fungi signal in the kidney in number 1 griffon. (b) Positive fungi signal in the kidney in number 2 griffon. (c) Positive fungi signal in the kidney in number 3 griffon. (d) Positive fungi signal in the kidney in number 4 griffon.

**Figure 4:**
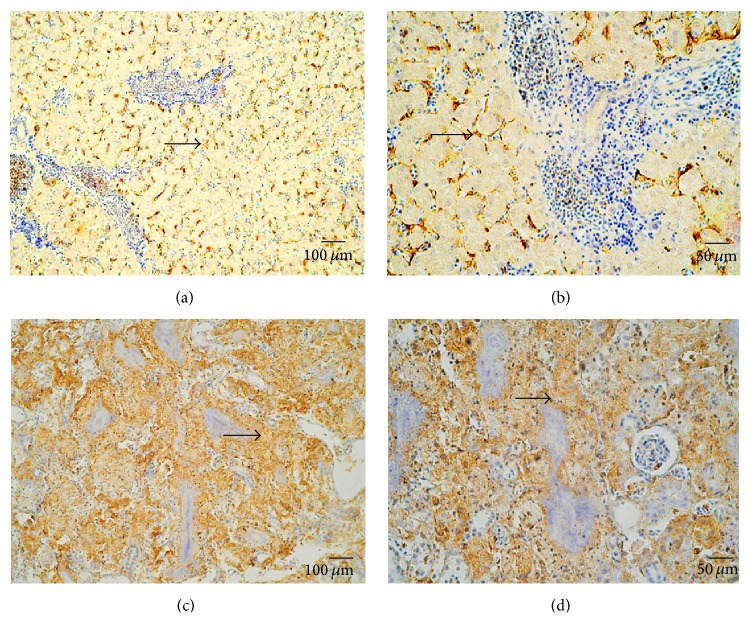
Detection of HEV antigens in the livers and kidneys of the dead griffons. (a) Positive HEV signal in the liver in number 1. (b) Positive HEV signal in the liver in number 2. (c) Positive HEV signal in the kidney in number 1. (d) Positive HEV signal in the kidney in number 2.

Periodic Acid-Schiff (PAS) stain confirmed the presence of fungi in the tissues and organs. The cell wall of the fungus contains rich polysaccharide, so PAS stain is easy to observe. Positive fungi signals were detected in the kidneys of all four griffons. A large number of purple-red fungal nodules were observed clearly in the kidneys (Figures 3(a), 3(b), 3(c), and 3(d)). We also tested HEV by Immunohistochemical (IHC) stain. HEV antigen was detected in the liver and kidney. Granular or diffuse positive staining was seen in the hepatic sinusoid and the cytoplasm of hepatocytes (Figures 4(a) and 4(b)). At the same time, the nuclei and cytoplasm of the renal tubular epithelial cells were positive for HEV antigen (Figures 4(c) and 4(d)).

### 3.4. Polymerase Chain Reaction Detection

**Figure 5:**
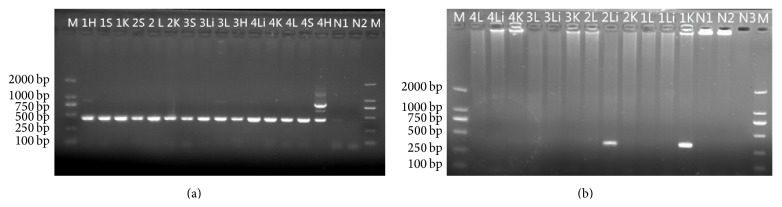
PCR assays of tissues with primers specific for fungi and HEV. (a) Fungi: lane M, DL2000 marker; 1, griffon 1 heart; 2, griffon 1 spleen; 3, griffon 1 kidney; 4, griffon 2 spleen; 5, griffon 2 lung; 6, griffon 2 kidney; 7, griffon 3 spleen; 8, griffon 3 liver; 9, griffon 3 lung; 10, griffon 3 heart; 11, griffon 4 liver; 12, griffon 4 kidney; 13, griffon 4 lung; 14, griffon 4 spleen; 15, griffon 4 heart; 16, negative control; 17, negative control; 18, lane M, DL2000 marker. The fungi amplicon was 425 bp. (b) HEV: positive for griffon 2 liver and griffon 1 kidney. The HEV amplicon was 348 bp.

**Figure 6:**
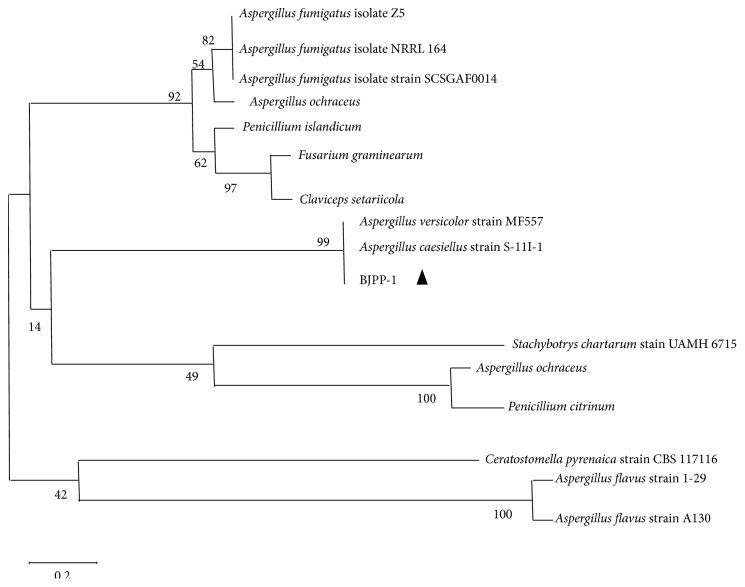
Phylogenetic analysis based on* Aspergillus* showing the genetic relationships between the isolates identified in this study. A neighbor-joining tree was constructed with bootstrap values calculated from 1,000 replicates. The isolates used for the comparative analysis were* Aspergillus fumigatus* Z5 (GQ337429),* Aspergillus fumigatus* NRRL 164 (JN850983),* Aspergillus fumigatus* SCGAF (EF669932),* Aspergillus ochraceus* (DQ336712),* Penicillium islandicum* (EU652685),* Fusarium graminearum* (AJ491293),* Claviceps setariicola* (FJ686007),* Aspergillus versicolor* MF557 (KM096354),* Aspergillus caesiellus* S-11I-1 (KM582669),* Stachybotrys chartarum* (AY180249),* Aspergillus ochraceus* (AB025476),* Penicillium citrinum* (AB041163),* Ceratostomella pyrenaica* (DQ076323),* Aspergillus flavus* 1-29 (AF036811), and* Aspergillus flavus* A130 (AF261857).

Using nutrient, MacConkey, and blood agar plates, there were no visible bacterial colonies in the liver, lung, heart, spleen, and kidney samples. We used polymerase chain reaction (PCR) techniques to determine the presence of pathogen. We detected NDV, FLU, HEV, and fungi 18SrRNA. NDV and FLU were negative in the livers, lungs, kidneys, and spleens. Nevertheless, the 18SrRNA results showed that fungi were detected in most organs (Figure 5(a)) and the sequencing results showed that it was an* Aspergillus* infection (Figure 6).

**Figure 7:**
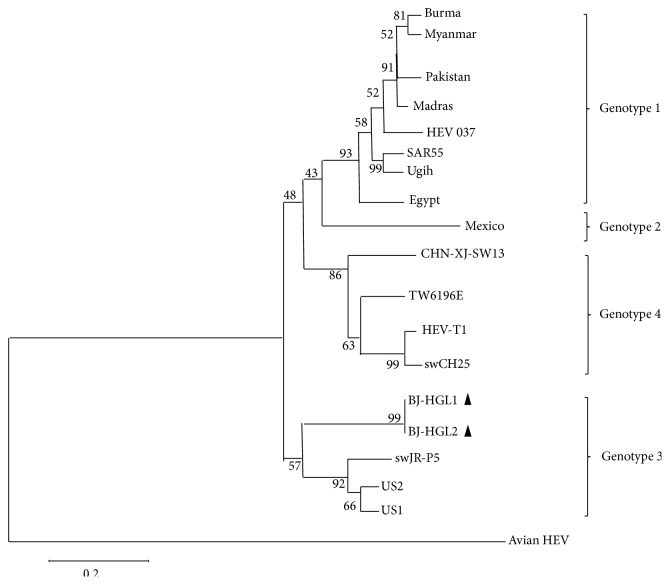
Phylogenetic analysis based on ORF2 (348 nt) depicting the genetic relationship between our isolates from this study. A neighbor-joining tree was constructed with bootstrap values calculated from 1,000 replicates. Isolates used for comparative analysis were Burma (GenBank Accession number M73218), Myanmar (D10330), Pakistan (AF185822), Madras (X99441), HEV 037 (X98292), SAR55 (M80581), Ugih (D11092), Egypt (AF051352), Mexico (M74506), CHN-XJ-SW13 (GU119961), TW6196E (HQ634346), HEV-T1 (AJ272108), swCH25 (AY594199), swJR-P5 (AB481229), US2 (AF060669), US1 (AF060668), and Avian HEV (AY535004).

At the same time, because of the serious pathological changes in the livers, we detected Avian HEV firstly. Unfortunately, the result of PCR was negative. However, we detect the presence of other types of HEV genotype in kidney and liver tissues (Figure 5(b)) (GenBank accession number KT002575). According to the neighbor-joining tree, the HEV isolates from the birds' kidney and liver were both a genotype III and were closely related to the swJR-P5 (AB481229), US2 (AF060669), and US1 (AF060668) (Figure 7).

We also did a PCR test for the environment and other birds manure. All samplings were negative for HEV detection. The 18SrRNA sequencing results showed that there are a certain number of fungi, such as* Penicillium*, in the surrounding environment. However, no potentially harmful* Aspergillus* fungus was detected in any other kinds of birds.

## 4. Discussion

Cases of Aspergillosis in birds are often diagnosed based on postmortem findings of white caseous nodules in the lungs and air sacs of affected birds [8]. However, there is a paucity of information on the observation of the fungal elements in the tissues of Himalayan griffons. In our study, many membranous-like patches and spots are observed in the kidney, liver, spleen, lung, intestine, and heart. Using H.E and PAS staining, we can see that there are many sunflower-like fungi nodes in these tissues, especially in the kidney. It indicated that* Aspergillus* could lead to multiple systemic infections in this case.

A BLAST search against the GenBank nr database showed 99% and 99% identities between the amplicon and the 18S ribosomal RNA regions of* A. versicolor* and* A. caesiellus*, respectively. Both of these two species belong to* Aspergillus* genus. Zhang et al. [21] once reported a case that* Aspergillus versicolor* could infect the canine and may be a new disseminated Aspergillosis. Like other members of* Aspergillus* species,* A. versicolor* is an opportunistic pathogen and is considered to be an important causative agent of Aspergillosis in the environment [22]. Meanwhile, there have been few literatures on* A. caesiellus* infection. However, whether Himalayan griffons are sensitive to* versicolor* or* caesiellus*, or whether small dose of the* Aspergillus* may lead to the renal infection, still requires animal regression experiments.

*Aspergillus* infecting birds were usually seen in the lungs. However, rare literature regarding the renal* Aspergillus* disease of Himalayan griffons was reported before. Barathidasan et al. [23] once reported an* Aspergillus* pneumonia of the Himalayan griffon. Fungal nodules were seen in the lung, air bag, pericardium, trachea, and the surface of pulmonary artery and aorta. Microscope observation showed necrosis inflammatory cell infiltration in lung. In our study, the infection route and pathological features are different from avian fungal pneumonia. Almost all tissues and organs showed severe lesions. The most obvious difference is the numerous renal* Aspergillus*. Areas of sunflower-like fungi nodes existed in renal interstitium. These fungi nodes' occupancy growth in kidney may lead to urinary system disorders and thereby affect the whole body metabolism. Maybe, this special phenomenon has some connection with the coinfection.

According to our observations, Immunohistochemical (IHC) stain and PCR detection for HEV showed positive results. Moreover, H.E staining showed severe liver lesions, such as hyperemia, necrosis, hemosiderosis, and focal lymphocytes infiltrating in the portal area. It indicated that two of the four Himalayan griffons were infected with HEV. Sequence results for this HEV strain showed that it belongs to genotype III. According to the literature report, HEV genotype III was known to be a zoonotic pathogen, which had been found in swine [16], rabbits [24], deer [25], mongooses [26], and any other wild animals. In our research, this HEV genotype III strain can be isolated from livers and kidneys. We assume it would be a cross-species infection. However, HEV has a long incubation period and the virus particles will vanish rapidly in the body. Immunity leads to virus clearance from the blood, while the virus may persist in stool for much longer [27]. So we detected HEV in only two birds while the other two birds' results were negative.

We assume that when animals were infected with HEV, their body immunity will decline. So fungi could invade the organism and cause infection. Without bladder, avian urinary system consists only of kidney and ureter. So this particular structure could also easily increase the chance of being infected.

## 5. Conclusion

According to the observation and results of PCR, We have excluded the possibility of the deposition of uric acid salt. And at the same time, this study provides important evidence for clinical diagnosis of the Avian Aspergillosis and Hepatitis E virus coinfection. Severe pathologic changes in these griffons indicated that fungi and virus coinfection might result in serious hepatic and renal consequences. However, whether these two pathogens interact still remains to be elucidated. Through the research of this study, we hope we can furnish a reference for the protection of wild rare animals and provide some ideas for future avian research.

## Figures and Tables

**Table 1:** Sequences of primers used in the PCR assays.

Primer	Sequence 5′-3′	Reference
16Sr DNA		[14]
16Sr DNA-F	AGAGTTTGATCCTGGCTCAG	
16Sr DNA-R	GGTTACCTTGTTACGACTT	
18SrRNA		[15]
FF2	GGTTCTATTTGTTGGTTTCTA	
FR1	CTCTCAATCTGTCAATCCTTATT	
HEV ORF2 gene		[16, 17]
HEV-externer primer	AATTATGCYCAGTAYCGRGTTG	
HEV-externer primer	CCCTTRTCYTGCTGMGCATTCTC	
HEV-interner primer	GTWATGCTYTGCATWCATGGCT	
HEV-interner primer	AGCCGACGAAATCAATTCTGTC	
Avian HEV helicase gene		[18]
AHEV F-1/SD	TGTTATT(C)ACACCCACCAAG(A)ACGT(C)TG	
Helic R-1	CCTCA(G)TGGACCGTA(T)ATCGACCC	
AHEV F-2/SD	GCCACGGCTG(A)TTACACCC(T)CAC(T)GT	
Helic R-2	GACCCA(G)GGA(G)TTCGACTGCTT	
Avian Influenza		[19]
Bm-fluA-F	TATTCGTCTCAGGGAGCAAAAGCAGG	
Bm-fluA-R	ATATCGTCTCGTATTAGTAGAAACAAGG	
M gene of NDV		[20]
AMPV1-F	AGTGATGTGCTCGGACCTTC	
AMPV1-R	CCTGAGGAGAGGCATTTGCTA	

**Table 2:** Main pathological changes in the organs of the four dead Himalayan griffons.

Organ	Pathological changes	1	2	3	4
		Fungus+	Fungus+	Fungus+	Fungus+
		HEV+	HEV+	HEV−	HEV−
Heart	Degeneration	+	+	+	−
	Edema	−	−	+	+
	Necrosis	+	+	+	+

Liver	Degeneration	+	+	+	+
	Edema	−	+	−	−
	Congestion	+	+	−	−
	Hemorrage	−	−	−	−
	Necrosis	+	+	−	+
	Inflammation	+	+	+	−

Lung	Degeneration	+	+	+	+
	Congestion	−	−	−	−
	Hemorrage	−	−	−	−
	Necrosis	+	+	+	+
	Fungus nodules	+	+	−	+

Kidney	Degeneration	+	+	+	+
	Edema	+	+	+	+
	Necrosis	+	+	+	+
	Fungus nodules	+	+	+	+

Spleen	Degeneration	+	+	+	+
	Edema	+	+	−	−
	Necrosis	+	+	+	+
	Fungus nodules	+	+	+	+

## Abbreviations

HEV: Hepatitis E virus
HE: Hepatitis E
NDV: Newcastle Disease Virus
FLU: Avian Influenza.
